# An immunohistochemical study of the localization and developmental expression of ghrelin and its functional receptor in the ovine placenta

**DOI:** 10.1186/1477-7827-5-25

**Published:** 2007-06-27

**Authors:** Joanne L Harrison, Clare L Adam, Yvonne A Brown, Jacqueline M Wallace, Raymond P Aitken, Richard G Lea, David W Miller

**Affiliations:** 1 School of Veterinary and Biomedical Sciences, Murdoch University, South Street, Murdoch, WA, Australia; 2 Obesity & Metabolic Health Division, Rowett Research Institute, Bucksburn, Aberdeen, UK; 3 School of Biological Sciences, University of Aberdeen, Aberdeen, UK; 4 Sustainable Livestock Systems Group, Scottish Agricultural College, Bucksburn, Aberdeen, UK; 5 School of Veterinary Medicine and Science, University of Nottingham, Sutton Bonington Campus, Leicestershire, UK

## Abstract

**Background:**

Ghrelin is an orexigenic hormone principally produced by the stomach, but also by numerous peripheral tissues including the placenta. Ghrelin acts via growth hormone secretagogue receptors (GHSR-1a) to alter food intake, fat utilization, and cellular proliferation, and has been suggested to play a role in the developmental growth of the fetoplacental unit. The placental expression of ghrelin and its role in ruminant species is not known. We tested the hypotheses that ghrelin and its functional receptor, GHSR-1a, are present in tissues of the ovine placenta, and that their expression is linked to the stage of development.

**Methods:**

Antibodies raised against ghrelin and GHSR-1a were used in standard immunohistochemical protocols on placental tissues collected from pregnant ewes (n = 6 per gestational time point) at days 50, 80, 100, 128 and 135 of gestation (term ≈ day 145). Immunostaining for ghrelin and GHSR-1a was quantified using computer-aided image analysis. Image analysis data were subjected to one-way ANOVA, with differences in immunostaining between time-points determined by Fisher's least significant difference.

**Results:**

Positive immunostaining for ghrelin was detected in ovine placentae at all gestational time points, with staining localized to the maternal epithelium, caruncle and trophectoderm. There was a significant effect of gestational age (p < 0.001) on the placental expression of ghrelin, with maximal levels at gestational day 80. GHSR-1a immunostaining was detected in the fetal trophectoderm at all time points. In contrast to the gestational pattern of ghrelin expression, there was no effect of gestational age on placental GHSR-1a immunoexpression.

**Conclusion:**

Ghrelin and GHSR-1a are both present in the ovine placenta, and ghrelin displays a developmentally-related pattern of expression. Therefore, these data strongly suggest that the ghrelin system may have a role in feto-placental development in sheep.

## Background

Recent studies have indicated a role for the orexigenic hormone, ghrelin, in the regulation of energy balance, food intake and body weight in monogastric species [1-4]. Ghrelin is also involved in the regulation of growth hormone (GH) secretion, being identified in 1999 as the endogenous ligand for the GH secretagogue receptor (GHSR-1a), a 7-transmembrane G protein coupled receptor [5]. In addition to its primary origin in the stomach [5,6], ghrelin and GHSR-1a have been found, in humans and rats, to have a wide distribution in other tissues, including the bowel, heart, kidney, liver, lung, pancreas, brain, gonads and placenta [7-9]. The significance of this wide tissue distribution has yet to be determined, however it has been suggested that ghrelin could be involved in the control of cellular differentiation, proliferation and apoptosis [10-15].

Although ruminant species also appear to utilise the ghrelin system to modulate endocrine and metabolic responses to nutrition and energy balance [6,16-21], little is known of the tissue distribution of ghrelin and its receptor, nor of its link to developmental processes in these species. Recently our group provided evidence to suggest that the expression of ghrelin and its receptor are developmentally regulated in the sheep fetus [22]. In adult animals, a tissue that undergoes marked development in a relatively short space of time is the placenta. In the placentae of humans and rats it has been suggested that ghrelin plays a role in development, growth and function of the fetoplacental unit due to the gestational-related changes in placental expression [7,23]. Based on umbilical vein and artery concentrations of ghrelin, Kitamura *et al*. [24] suggested that a proportion of ghrelin in the circulation of the human fetus might originate from the placenta and act to regulate feto-maternal energy transport and growth. In addition, ghrelin levels in the late gestation rat fetus are unaffected by food restriction of the dam [25] even though food restriction is known to affect maternal plasma ghrelin levels [26]. It would appear that the late gestation fetus is buffered, perhaps via a dominant placental or fetal source of ghrelin, against maternal changes in ghrelin levels. However, the exact contribution and ontogeny of maternal versus feto-placental sources of ghrelin to circulating fetal concentrations is unclear. The sheep provides a good animal model for investigating the possible role of ghrelin in human placental development and function because, unlike rodent models, the temporal changes of placental growth are similar in length to the human, but unlike humans, there are no strict ethical constraints on obtaining mid to late gestation ovine placental tissue.

In the present study we used immunohistochemistry to test the hypothesis that ghrelin and its functional receptor, GHSR-1a, are present in tissues of the ovine placenta. In addition, we examined the ontogeny of ghrelin and GHSR-1a immunoreactivity in the developing placenta throughout gestation.

## Materials and methods

### Tissue collection and processing

All experimental procedures involving animals were conducted under the authority of the Animals (Scientific Procedures) Act, UK, 1986 after Home Office and local ethical committee approval. Singleton pregnancies in Suffolk x Dorset Horn x Greyface ewes were established by embryo recovery and transfer procedures as described by Wallace *et al*. [27], using the same individual Dorset Horn sire. This technique removes the potentially confounding influence of partial embryo loss and variation in fetal number, and maximises the homogeneity of the resulting fetuses. Placental tissues from ewes, nutritionally managed to exhibit normal placental and fetal growth, were collected at autopsy on days 50, 80, 100, 128 and 135 of gestation (term ≈ day 145). These tissues (n = 6 per gestational time point) were collected as 'control' tissues from a number of individual experiments, but all using the same procedures and animals as described above [28-31]. The embryo donor and recipient genotypes were identical between groups, as were the age, parity and nutritional management of the recipient dams. A representative placentome from each animal was collected for processing for the immunohistochemical analyses. The selection of the placentome for immunohistochemistry was based on the picking the median sized placentome once the range in placentome sizes for each individual animal had been ascertained. Whole placentomes were sliced into 5 mm cross-sections and immersion fixed in normal buffered formalin (NBF) for 24 h. Samples were then rinsed and stored in 70% alcohol before processing and embedding into paraffin wax. Sections (5 μm) were cut and mounted on polylysine coated glass slides and dried overnight at 42°C prior to immunohistochemical analysis.

### Placental growth curve

**Figure 3:**
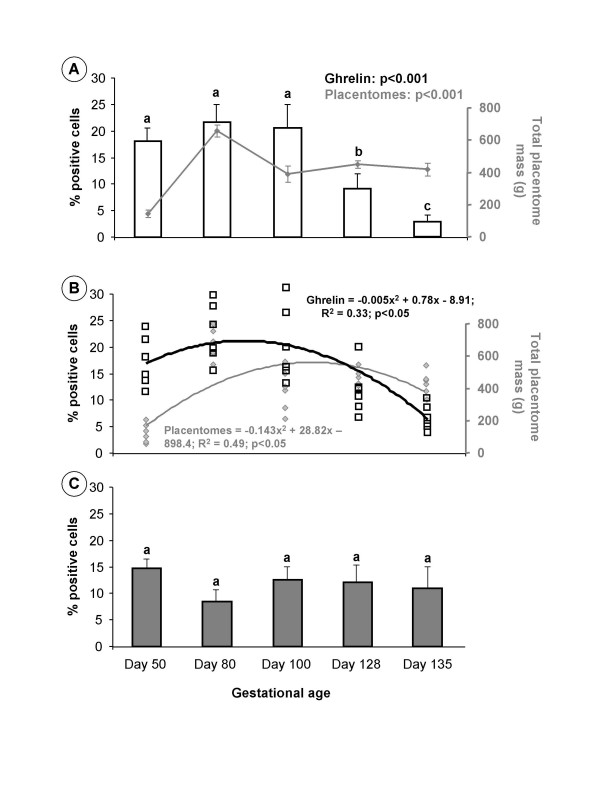
**Effect of gestational age on placentomes mass and immunoexpression of placental ghrelin and growth hormone secretagogue receptor (GHSR)-1a**. Effect of gestational age on the percentage of cells positively immunostained for ghrelin (a) and GHSR-1a (c) in sheep placentomes collected between days 50 and 135 of gestation (n = 6 at each gestational age). In Fig. 3a the change in total placentome mass is superimposed as the grey line on the graph for ghrelin, and in Fig. 3b the comparison is shown for the 2^nd ^order polynomial lines of best fit with line equations for the gestational change in ghrelin (black text equation, white squares, black line) and total placentomes mass (grey text equation, grey diamonds, grey line) (b). Values with different alphabetical superscripts are statistically significant compared to the peak (ghrelin) and nadir (GHSR-1a) levels at day 80: a versus b = p < 0.05; a versus c = p < 0.01; a versus d = p < 0.001. Significant changes within variables over time are annotated to each figure. Values are means ± S.E.M.

Total placentome weight, with the fetal cotyledon and maternal caruncle intact, was recorded for each animal at each gestational time point, after carefully separating out the individual placentomes from the placental mass [28-31]. These data were used to construct the placental growth curve represented in Figure 3.

### Immunohistochemistry

The immunohistochemistry methods have been previously described by Miller *et al*. [22]. Briefly, tissue sections were dewaxed in Histoclear (National Diagnostics, Hessel, Hull, UK), rehydrated through a graded ethanol series (100%, 95% and 70%). Antigen retrieval procedures were necessary for exposure of all epitopes, and this was achieved by microwaving sections in 0.01 M citrate buffer (pH 6.0) on full power for 3 × 5 min. To remove possible variation arising from manual staining techniques, placental sections were placed in an Autostainer (Dako, Ely, UK) at room temperature and incubated with the appropriate primary antibodies, as follows: (a) anti-human ghrelin (Cat. H-031-30: Phoenix Europe GmbH, Karlsruhe, Germany) at a 1:600 dilution for 30 mins, and (b) anti-human GHSR-1a Cys^0 ^(330–366) (Cat. H-001-62: Phoenix Europe GmbH, Karlsruhe, Germany) at a 1:600 dilution for 30 minutes. The negative controls were produced by substituting the primary antibody with normal rabbit serum (ghrelin) or normal goat serum (GHSR-1a) at the same dilution as the primary antibody. Ghrelin detection was done using the DAKO ChemMate perioxidase/DAB system (DakoCytomation Ltd, Ely, UK) and GHSR-1a used the Vectastain Elite ABC system (Vector Laboratories Ltd, Peterborough, UK). Antibody binding was visualised by the ChemMate peroxidase/DAB detection system (DakoCytomation Ltd), applied in two 5 minute incubations using the Dako autostainer, and all sections were counterstained with haematoxylin Z (Cellpath, Hemel Hampstead, UK).

To check antibody specificity, both primary antibodies were incubated overnight with their respective immunizing peptides. Following incubation, antibody-antigen preparations were centrifuged and the supernatant applied to selected placental tissue sections. We have previously published verification methods and data for the specificity of both antibodies in stomach (abomasum) and pituitary tissue from sheep, along with *in-situ *hybridization which revealed similar mRNA localization to the peptide immunoexpression [22].

### Quantification by image analysis

The expression of ghrelin and GHSR-1a was analyzed using computer-aided image analysis. The system was composed of a Ziess axioplan microscope (Zeiss, West Germany) and a HV-C20 Hitachi camera (Hitachi, Japan) connected to a computer running Image-ProPlus software (Media Cybernetics, Maryland, USA). Sections were quantified for percentage of cells that were immunopositive (brown colour) over ten randomly selected fields of view, which was sufficient replication to stabilise the mean and standard error, as previously described by Murray *et al*. [32]. The number of positively stained cells (brown) was selected and expressed as a sum of pixels. All nuclei were then selected and then the positively stained cells were expressed as a percentage of the total.

### Statistical analyses

Image analysis data were subjected to ANOVA with post-hoc differences between placental immunostaining intensity for ghrelin and GHSR-1a at different stages of gestation determined by Fisher's least significant difference. Correlation between total placentome mass and ghrelin expression or GHSR-1a expression was investigated by simple regression analysis.

## Results

### Antibody specificity

**Figure 1:**
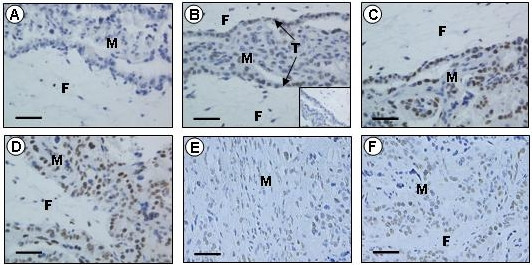
**Immunohistochemical localization of ghrelin in the sheep placenta**. Representative immunohistochemical photomicrographs showing localization of ghrelin (b – f) in sheep placentomes collected at days 50 (b), 80 (c), 100 (d), 128 (e) and 135 (f). The insert in the bottom right corner (b) represents an example of a negative control. Positive immunostaining in the placenta was abolished (a) when the antiserum was pre-incubated with the immunizing peptide (ghrelin). Placentome sections mainly show positive immunostaining for ghrelin (brown) in the maternal compartment (M) and the trophectoderm (T), with few cells being immunopositive for ghrelin in the fetal compartment (F). The scale bars represent 50 μm.

**Figure 2:**
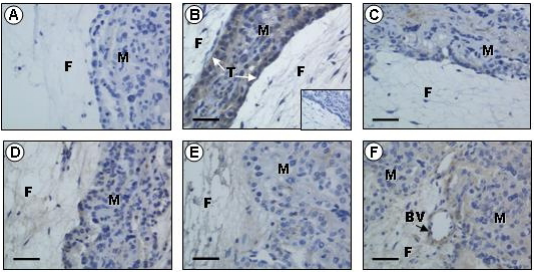
**Immunohistochemical localization of growth hormone secretagogue receptor (GHSR)-1a in the sheep placenta**. Representative immunohistochemical photomicrographs showing localization of GHSR-1a (b – f) in sheep placentomes collected at days 50 (b), 80 (c), 100 (d), 128 (e) and 135 (f). Insert (b) represents an example of a negative control. Positive immunostaining in the placenta was abolished (a) when the antiserum was pre-incubated with the immunizing peptide (GHSR-1a). Placentome sections mainly show positive immunostaining for GHSR-1a (brown) in the maternal compartment (M) and the trophectoderm (T), and in the blood vessels (BV). The scale bars represent 50 μm.

The specificity of the two antibodies (anti-ghrelin and anti-GHSR1a) was confirmed by the absence of immunostaining when the antisera were pre-incubated with the respective immunizing peptides (Figs. 1a &2a). Additionally, immunostaining was abolished in the controls when the primary antibodies were replaced with serum from the species in which the antibodies were raised (inserts: Figs. 1b &2b). In all tissues investigated, the ghrelin immunopositive cells exhibited strong perinuclear staining and/or more disperse cytoplasmic staining (Fig. 1). The GHSR-1a positive cells also exhibited some perinuclear staining, but mainly cytoplasmic staining (Fig. 2).

### Ghrelin and GHSR-1a immunolocalization in placental tissues

Ghrelin immunoreactivity was detected in the ovine placental tissues at all of the gestational time points examined, with immunopositive staining localized to the maternal epithelium, caruncle and the trophectoderm (Fig. 1b–f). Placental GHSR-1a was detected mainly in the fetal trophectoderm at all of the gestational time points examined (Fig. 2b–f). Additionally, the blood vessels of the ovine placenta were also immunopositive for GHSR-1a (Fig. 2f).

### Ghrelin and GHSR-1a immunoexpression throughout gestation

There was a significant effect of gestational age (p < 0.001) on the percentage of cells showing positive immunoreactivity for ghrelin (Fig. 3a). Placental ghrelin immunoreactivity was maximal between day 50 to 100 of gestation, reaching peak levels at day 80. At day 128, placental ghrelin levels had decreased to less than half of the peak level observed at day 80 (p < 0.01). Ghrelin immunostaining continued to decline to day 135, the final gestational point used in this study, with observed ghrelin levels being less than 10% of the peak levels at day 80 (p < 0.01). In comparison, there was no effect of gestational age on placental GHSR-1a immunoexpression (Fig. 3c).

### Placental growth throughout gestation

The change in total placentome mass with gestational age corresponded to the changes in the percentage of cells staining immunopositive for ghrelin (Fig. 3a). There was a significant effect of gestational age (p < 0.001) on the total placentome mass, which was minimum at day 50 (143 ± 22.5 g) and peaked at day 80 (657 ± 35.9 g), before declining again at day 100 (387 ± 50.3 g) and remaining at this lower level till day 135 (414 ± 40.8 g). There was a significant correlation (R^2 ^= 0.51; p < 0.05) between the gestational change in total placentome mass and ghrelin immunoexpression, represented graphically by comparison of the 2^nd ^order polynomial lines of best fit for the two data sets (Fig. 3b). There was no correlation between the gestational change in total placentome mass and GHSR-1a immunoexpression.

## Discussion

The present study provides immunohistochemical evidence for the presence of ghrelin and its functional receptor, GHSR-1a, in placental tissues of the sheep. Novel data are also presented that indicate that levels of ghrelin, but not GHSR-1a, are regulated during development of the ovine placenta. The presence of both components (ligand and receptor) of the ghrelin signalling system in the ovine placenta, and the changes in developmental immunoexpression, are consistent with a putative role of ghrelin in placental development and function in this ruminant species. However, it must be noted that ghrelin 'knockout' mice are fertile and do produce normal litters [33]. However, knockout models sometimes indicate, as may be the case here, that there are multiple systems for controlling critical developmental process such as feto-placental growth.

In rats and humans, Gualillo *et al*. [23] also demonstrated the presence of ghrelin mRNA and protein in the placenta, and that expression levels decreased as the placentae developed past its peak proliferative phase. These results, together with the present findings, strongly indicate that the placenta is a significant source of ghrelin during mid-gestation, and that the ghrelin ligand-receptor system may play a role in development of the feto-placental unit in both ruminant and non-ruminant mammals. Maternal ghrelin levels are increased in pregnant rats that are food-restricted from mid-pregnancy resulting in intrauterine growth-restricted (IUGR) offspring [34]. Studies also suggest that umbilical cord ghrelin levels are higher in human small-for-gestational-age (SGA) fetuses [35,36], yet there is no correlation between fetal and maternal ghrelin levels in the SGA condition [37]. Bellone *et al*. [38] found that ghrelin levels in human newborns are higher than those in their mothers indicating significant feto-placental ghrelin production. In early pregnancy, ghrelin has been found to inhibit the development of mouse embryos [39], but in late pregnancy chronic treatment of rat mothers with ghrelin increases birth weight of the offspring, and rat mothers immunized against ghrelin deliver fetuses with lower body weights [40]. Perhaps in early gestation, high plasma concentrations of maternally-derived ghrelin that would occur during states of malnutrition may act as an 'embryonic termination' signal to circumvent the excess metabolic demands of pregnancy, but in mid to late gestation placenta-derived ghrelin in the normal concentration range may be essential for the growth and development of the fetus. Further research is required to determine the validity of these postulates.

In the present study, ovine placental ghrelin peptide expression was maximal at gestational days 50 to 100 and decreased to late gestation (day 135; NB: term ≈ day 147). This observation coincided with gestational-age related changes in total placentome mass calculated in the present study, and also agrees with changes in placental growth and cell proliferation in sheep gathered by other researchers [41,42]. Peak ghrelin peptide expression therefore corresponds to the active proliferation phase of the placenta. Previously, we have demonstrated an association between ghrelin immunoexpression and immunohistochemical markers of cell proliferation in the ovine fetal testis [22]. Therefore, it is tempting to speculate that ghrelin may be influencing growth of the feto-placental unit by affecting cellular proliferation. However, data on the effects of ghrelin upon cell proliferation are equivocal with positive effects observed in adipocyte, cardiomyocyte, osteoblast and pituitary cell lines [12,13,43,44], and negative effects seen in breast, lung and thyroid carcinoma cell lines [45-47]. Further studies of this postulated effect of ghrelin on placental growth and proliferation obviously needs to be conducted as perturbation of these facets of the feto-placental unit could contribute to developmental origins of adult disease [48]. For example, placental size and the ratio of placental weight to birth weight have been found to be associated with type 2 diabetes and impaired glucose tolerance in humans [49].

Ghrelin could be involved in the local modulation of placental GH release, which is known to affect intrauterine development in sheep [31,50-52]. However, Fuglsang *et al*. [53] found no associations between maternal ghrelin levels and placental GH production in humans, and Kitamura *et al*. [24] found that plasma ghrelin concentrations in human cord blood were not correlated with GH concentration, but they were correlated to IGF-I concentrations. Alternatively, placental ghrelin could influence maternal and/or fetal pituitary GH secretion. The administration of exogenous GH increases placental and fetal growth, whereas a GH deficiency is associated with fetal growth retardation [54]. Therefore, it could be hypothesized that placental derived ghrelin may influence fetal growth indirectly through modulation of pituitary GH secretion.

Particularly evident in the sheep placental tissue was the finding that ghrelin immunoreactivity in some cells showed intense perinuclear staining, in cases resembling nuclear staining. This is consistent with a previous study by our group that also showed that the nuclei were indeed immuno-negative when observed at high magnification [22]. This perinuclear staining pattern may arise if the polyclonal antibody is detecting the ligand/receptor complex and is consistent with the finding of Camina *et al*. [55] that the ghrelin/GHSR-1a complex progressively disappears from the plasma membrane after binding of the ligand and accumulates in the perinuclear region. Additionally, GHSR-1a showed positive staining in blood vessels of the ovine placenta, in agreement with GHSR-1a expression in adult and fetal testicular blood vessels in a previous study [22]. It has previously been suggested that the ghrelin ligand/receptor complex plays a role in apoptotic control of endothelial cells through ERK1/2 and PI 3-kinase/AKT pathways [11].

Finally, placental ghrelin may have an immunomodulatory role in pregnancy. For a successful pregnancy, it is well recognised that a state of selective tolerance, immunosuppression and immunomodulation in the presence of strong anti-microbial immunity must be established [56]. Maternal adaptation to pregnancy involves down regulation of potentially dangerous T-cell mediated immune responses and activation of components of the innate immune system, such as monocytes and neutrophils [57]. Hattori *et al*. [58] found both ghrelin and GHSR expression in human immune cells. They also found expression of this peptide and ligand in B-cells, T-cells and neutrophils that did not express substantial GH transcripts suggesting that ghrelin/GHSR has biological functions other than enhancing GH secretion in the immune system. Wu and Kral [59] found that ghrelin enhances immune responses and potentially down-regulates anti-inflammatory molecules. Recently Dixit *et al*. [60] demonstrated that ghrelin, via functional cell surface GHSR, exerts both selective inhibitory effects on the expression and production of the inflammatory cytokines IL-1β, IL-6, and TNF-α by human PBMCs and T cells. Obviously, more research is needed to elucidate whether any of these putative actions of ghrelin, perhaps of a placental source, may play a role in immunomodulation during pregnancy.

## Conclusion

This study has demonstrated for the first time that both ghrelin and its receptor, GHSR-1a, are present in ovine placental tissue. Additionally, by sampling at several gestational windows we have shown that maximum and minimum placental ghrelin immunoexpression coincides with the time of maximum and minimum placental growth in the sheep, respectively. This suggests a possible role for ghrelin in the growth and/or function of the ovine placenta and/or fetus.

## Competing interests

The authors declare that they have no competing interests that would prejudice the impartiality of this scientific work.

## Authors' contributions

All authors have read and approved the final manuscript. JLH participated in the design of the studies, collection of tissues, immunological analyses of ghrelin and GHSR-1a, statistical analyses and drafting the manuscript. CLA participated in the design of the studies, collection of tissues, statistical analyses and drafting the manuscript. YAB participated in the immunological analysis of GHSR-1a, statistical analysis and drafting the manuscript. JMW participated in the design of the studies, collection of tissues and drafting the manuscript. RPA participated in the design of the studies and collection of tissues. RGL participated in the design of the studies, all immunological analyses, statistical analyses and drafting the manuscript. DWM participated in the design of the studies, collection of tissues, all immunological analyses, statistical analyses and drafting the manuscript.
